# Deafness Cured by Excision of Tonsils

**Published:** 1886-10

**Authors:** J. N. Martin

**Affiliations:** Lecturer on Oral Diseases, Dental Department, in University of Michigan


### Deafness Cured by Excision of Tonsils.

(J. N. Martin, M.D., Lecturer on Oral Diseases, Dental Department, in University of Michigan.)

Ann Arbor, Aug. 24, 1886.

The close relation existing between the tonsils and jaws and their appendages; the jaws, etc., and the ear; the tonsils and the ear, affords a field of no little interest and importance to dentists and physicians; the phenomena resulting in the aural structures from disease in the tonsils, jaws, and teeth are numerous, and too often the outermost branches of the manifestations of disease are treated while the great trunk, the seat of disease, is neglected.

It is a well-known fact by aurists that many so-called diseases of the ear come from diseases of the oral structures, and are really symptoms and signs of disease in other localities; diseases of the oral structures by reflex action often produce otalgia or ear-ache. Of several cases that have come under my care illustrating the above named relation, I will present one which will, I trust, be of value to some of the readers of the REGISTER:

Patient, Mr. D., aged 22, Canton, Ohio, came to me for treatment April 6, 1886, for "throat trouble." Briefly, the history of the case is the following:

As long as patient could remember he had been afflicted with "ear trouble;" all of the time his hearing was somewhat impaired, but at times much worse; he had had attacks of "sore throat" frequently during this time, and not infrequently tonsilitis; his hearing always more impaired, while his throat was sore; and generally ear-ache accompanied these attacks of sore throat. His general health had always been good.

When patient applied for treatment an examination revealed acute pharyngitis and some inflammation of the tonsils; tonsils somewhat swollen, one greatly hypertrophied and the other somewhat so; I treated his throat until April 17, when, the acute inflammation having disappeared, I advised excision of the tonsils, believing that their hypertrophied condition was the cause, as well as the result, of the repeated attacks of tonsillitis and pharyngitis; *also that the swelling and thickening of the tissues about the openings of the Eustachian tubes, resulting from these inflammations, is a fruitful cause of impairment of the aural organs.*

I excised both tonsils April 17 and patient's hearing was improved within a few hours; two weeks later his hearing was nearly perfect; on June 7, 1886, his hearing was perfect and throat perfectly well, without an attack of sore throat since the operation.

---

A CIGAR contains acetic, carbolic, formic, butyric, valeric, prussic, and propionic acids, also creasote, ammonia, sulphuretted hydrogen, pyridine, viridine, picoline, and rubidene, to say nothing of cabbagine and burdockic acid. That's why you can't get a good one for less than five cents.

